# Expression and prognostic significance of THBS1, Cyr61 and CTGF in esophageal squamous cell carcinoma

**DOI:** 10.1186/1471-2407-9-291

**Published:** 2009-08-22

**Authors:** Zhu-Qing Zhou, Wei-Hua Cao, Jian-Jun Xie, Jing Lin, Zhong-Ying Shen, Qing-Ying Zhang, Jin-Hui Shen, Li-Yan Xu, En-Min Li

**Affiliations:** 1 Department of Biochemistry and Molecular Biology, Shantou University, Shantou, PR China; 2 Institute of Oncologic Pathology, Shantou University, Shantou, PR China; 3 Department of Hospice Care of the First Affiliated Hospital, Shantou University, Shantou, PR China; 4 Department of Public Health and Preventive Medicine, Medical College of Shantou University, Shantou, PR China; 5 Department of Clinical Pathology, Centre Hospital of Shantou City, Shantou 515041, PR China

## Abstract

**Background:**

Thrombospondin1 (THBS1), cystene-rich protein 61 (Cyr61) and connective tissue growth factor (CTGF) are all involved in the transforming growth factor-beta (TGF-β) signal pathway, which plays an important role in the tumorigenesis. The purpose of this study is to explore the expression and prognostic significance of these proteins in esophageal squamous cell carcinoma (ESCC).

**Methods:**

We used immunohistochemistry and western blotting to examine the expression status of THBS1, Cyr61 and CTGF in ESCC. Correlations of THBS1, Cyr61 and CTGF over-expressions with various clinicopathologic factors were also determined by using the Chi-square test or Fisher's exact probability test. Survival analysis was assessed by the Kaplan-Meier analysis and the log-rank test. Relative risk was evaluated by the multivariate Cox proportional hazards model.

**Results:**

THBS1, Cyr61 and CTGF were all over-expressed in ESCC. THBS1 over-expression was significantly associated with TNM stage (*P *= 0.029) and regional lymph node involvement (*P *= 0.026). Kaplan-Meier survival analysis showed that over-expression of THBS1, Cyr61 or CTGF was related to poor survival of ESCC patients (*P *= 0.042, *P *= 0.020, *P *= 0.018, respectively). Multivariate Cox analysis demonstrated that Cyr61 and CTGF were independent factors in prognosis of ESCC.

**Conclusion:**

Cyr61, CTGF and THBS1 were all over-expressed in ESCC and might be new molecular markers to predict the prognosis of ESCC patients.

## Background

Esophageal squamous cell carcinoma (ESCC) is the fourth most common malignancy in China, even prevalent throughout the world, with high mortality rate [1]. Diagnosis of ESCC at its early stage still remains difficult and advanced ESCC frequently displays local invasion and lymph node metastasis, which is one of the important reasons for its poor prognosis [2]. The development of this malignancy rises from the stepwise accumulations of multiple genetic alterations, leading to the activation of oncogenes and/or the inactivation of tumor suppressor genes.

Transforming growth factor-beta (TGF-β) is both a tumor suppressor and tumor oncogene [3]. Early in carcinogenesis it acts as a tumor suppressor but later it acts as a stimulator of tumor invasion by prompting extracellular matrix production and angiogenesis, stimulating tumor proliferation, and inhibiting host immune functions [4-6]. TGF-β signaling pathway can be activated by thrombospondin1 (THBS1) through its interaction with latent TGF-β binding proteins (LTBP) so that TGF-β is capable of binding to its receptors and stimulating the Smad pathway [7,8]. Smad proteins are able to bind to cystene-rich protein 61 (Cyr61) and connective tissue growth factor (CTGF) promoters, which leads to transcription of Cyr61 and CTGF and activation of angiogenesis and tumor growth [9,10].

Depending on the cell type, THBS1, CTGF and Cyr61 may have both negative and positive effect on tumor progression [11,12]. It has been reported that THBS1 expression in the stroma of ESCC was correlated with lymph node metastasis and Cyr61 expression in Barrett's tissue of esophageal adenocarcinoma was significantly higher than that in Barrett's esophageal tissue with no sign of cancer [13,14]. Recently, CTGF expression was found to be up-regulated in ESCC and significantly related to survival of ESCC patients [15,16]. However, the expressions and the prognostic significances of Cyr61 and THBS1 in ESCC still remain uncertain.

To further explore the expression patterns of THBS1, Cyr61 and CTGF and determine whether these proteins could be prognostic factors in ESCC, we investigated their expressions in a series of ESCC by using immunohistochemistry staining and western blotting. Furthermore, the correlations of these proteins with survival of ESCC patients were also addressed.

## Methods

### Patient selection

Eighty surgically removed ESCC samples (complete tumor resection, R0) embedded in paraffin wax blocks and thirty-eight cases of para-cancerous epithelium, collected between 1997 and 2006, were retrieved from the Department of Clinical Pathology at Center Hospital of Shantou City, Tumor Hospital of Shantou University Medical College and the Second Affiliated Hospital of Shantou University Medical College. The cases (only surgery) were selected in this study if a follow-up was obtained and clinical data were available. This study was approved by the ethical committee of the Center Hospital of Shantou City, the ethical committee of Tumor Hospital of Shantou University Medical College and the ethical committee of the Second Affiliated Hospital of Shantou University Medical College. The mean age of the patients was 57 years (range: 36–75). These paraffin blocks had undergone TMA construction before immunostaining. Paraffin sections were directly subjected to pathological staining and immunohistochemistry. At the end of the follow-up period there were no surviving patients.

### Construction of tissue microarrays

The construction of esophageal carcinoma tissue microarray was performed as described before [17]. Briefly, representative regions of each tissue were selected from hematoxylin- and eosin-stained sections and marked on the individual paraffin blocks. Samples were chosen from those specimens with more tissue available, so that correlative studies would not be compromised. Two tissue cores were obtained from each specimen, measuring 1.8 mm in diameter and ranging in length from 1.0 to 3.0 mm depending on the depth of tissue in the donor block. Each core was precisely arrayed into a new paraffin block. These microarrays were serially sectioned (4 μm), and stained with hematoxylin and eosin to verify tissue sampling and completeness. The unstained sections were baked overnight at 56°C in preparation for immunohistochemistry.

### Immunohistochemical evaluation

Mouse monoclonal CTGF antibody (1:100 dilution in PBS containing 0.01% Triton X-100, R&D Systems, USA), rabbit polyclonal Cyr61 antibody (1:100 dilution, Novus Biologicals) and mouse monoclonal THBS1 antibody (1:20 dilution, R&D Systems) were used in this study. The superPicTure Polymer Detection Kit and the Liquid DAB Substrate Kit (Zymed/Invitrogen) were used to carry out the immunohistochemical studies. Briefly, slides were submerged in a Peroxidase Quenching Solution, containing 1 part of 30% hydrogen peroxide to 9 parts of absolute methanol, for 10 minutes and were then washed with PBS. Then, 100 μl of serum blocking solution was added to each section, followed by incubation for 10 min and then draining the solution. At this time, 100 μl of the appropriate antibodies were applied to each section, and the slides were incubated in a moist chamber for 30 min, followed by a rinse with PBS. After rinsing, 100 μl of HRP Polymer Conjugate was applied to each section, and incubated for 10 min, followed by a rinse with PBS. Finally, 100 μl of DAB chromogen was applied to each section and incubated for 3–10 min. Samples were then rinsed well with distilled water. Subsequently, slides were counterstained with Maye's hematoxylin, dehydrated, and mounted. The negative controls were prepared by substituting PBS for the primary antibodies.

Staining of the cores was scored based on signal intensity (0–3) and the percentage of positive cells (0 ≤ 10%, 1 = 10–25%, 2 = 25–50%, and 3 ≥ 50%). Over-expressions of THBS1, Cyr61 and CTGF were arbitrarily defined as more than 25% of cells with strong staining (score 2 and 3). Input from visual inspection data and detection of events in digitized images was stored in dedicated tables for comparison and statistical analysis. A disc was considered unsuitable for analysis if it was completely absent, if it contained no tumor tissue (sampling error), or if it contained too few (<10%) tumor cells for analysis (uninformative). For each patient, the mean score of a minimum of two core samples was calculated.

### Western blotting analysis

Tissues were lysed in a RIPA buffer and the protein concentration was estimated by the Bradford method. Equal amounts of tissue or cell lysates (50 μg) were electrophoresed on 10% polyacrylamide gel and transferred to polyvinylidene difluoride membranes (Millipore). The membraneswere then blocked with 5% skim milk-phosphate-buffered saline Tween (0.01 M PBS, 0.05% Tween 20) for 1 h and incubated at room temperature for 1 h with the primary antibody as described above. The membrane was subsequently incubated at room temperature for 1 h with horse radish peroxidase-linked second antibody and analysed using western blotting Luminol Reagent (Santa Cruz Biotechnology). Image acquisition and quantitative analysis were carried out using the FluorChem 8900 image analysis system (Alpha Innotech).

### Statistical analysis

Correlations of THBS1, Cyr61 and CTGF over-expressions with the clinical and pathological variables including gender, regional lymph node involvement, histological grade and TNM stage were made using the Chi-square test or Fisher's exact probability test. Survival was assessed by Kaplan-Meier analysis with log-rank score for determining statistical significance. Relative risk was evaluated by the multivariate Cox proportional hazards model. Calculation and analysis were performed with SPSS for Windows (Version 13.0) and where appropriate, was two tailed. *P *value less than 0.05 was considered as statistically significant.

## Results

### Clinical and pathological variable analysis

Samples from eighty patients who underwent primary surgical resection for ESCC met inclusion criteria were used to build tissue microarray. Of the esophageal squamous cell carcinoma that met inclusion criteria, all were interpretable for THBS1, Cyr61 and CTGF staining. Demographic and clinicopathological variables for the cohort were summarised in Table 1.

**Table 1:** Comparison of Cyr61, CTGF and THBS1 immunohistochemisty wih clinicopathological features in patients with ESCC

	THBS1^†^				Cyr61^†^				CTGF^†^			
	n	-	+	*P*	n	-	+	*P*	n	-	+	*P*
**Age(years)**												
≤ 57	43	6	37	0.494	43	21	22	0.987	43	25	18	0.184
>57	37	3	34		37	18	19		37	16	21	
**Gender**												
Male	58	6	52	0.700	58	29	29	0.805	58	33	25	0.101
Female	22	3	19		22	10	12		22	8	14	
**Regional lymph node**												
N0	48	2	46	0.026*	48	23	25	0.855	48	27	21	0.273
N1	32	7	25		32	16	16		32	14	18	
**Primary tumor**												
T1	4	1	3		4	2	2		4	3	1	
T2	6	0	6		6	3	3		6	2	4	
T3	64	7	57	0.479	64	30	34	0.868	64	34	30	0.501
T4	6	1	5		6	4	2		6	2	4	
**Histologic grade**												
Well differentiated	24	1	23		24	11	13		24	11	13	
Moderately differentiated	48	6	42	0.179	48	22	26	0.355	48	26	22	0.844
Poorly differentiated	8	2	6		8	6	2		8	4	4	
**TNM Stage**												
I/IIa	45	1	44		45	21	24		45	26	19	
IIb/III	35	8	27	0.009*	35	18	17	0.673	35	15	20	0.185
**ESCC**	80	8	72		80	39	41		80	41	39	
**Normal mucosa**	38	31	7	0.000*	38	34	4	0.000*	38	30	8	0.004*

TNM: tumor node metastasis; ^†^: (-) = nonoverexpressor; (+) = overexpressor.

*Significant at *P *< 0.05 level.

### Expressions THBS1, Cyr61 and CTGF in ESCC

**Figure 1:**
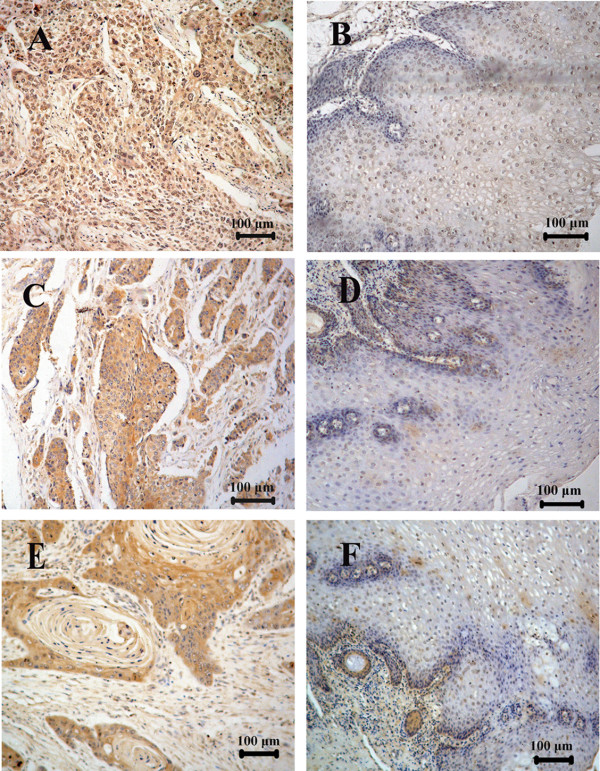
**Expressions of THBS1, Cyr61 and CTGF in normal human esophageal epithelium and ESCC**. Staining of the three genes was performed as indicated in Materials and Methods. Scale bar represents 100 μm in the tissue core shown in panel A-F. THBS1 (A), Cyr61 (B) and CTGF (C) in ESCC demonstrated strong cytoplasmic staining which was scored as over-expression. THBS1 (B), Cyr61 (C) and CTGF (D) in normal esophageal epithelium showed weak or absent staining and were scored as non-overexpression.

**Figure 2:**
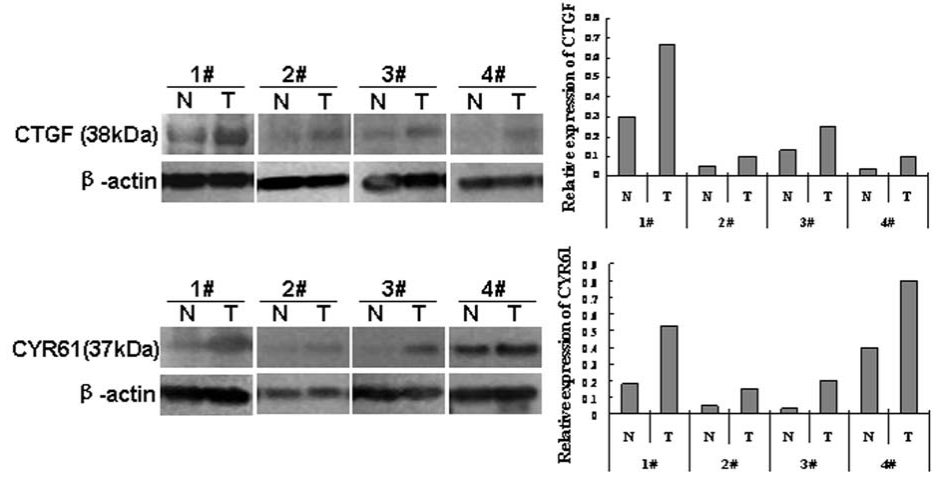
**Expressions of CTGF and CYR61 protein in four, randomly picked, paired ESCC samples were analyzed by western blotting**. Signal intensities for the expression of p-ERK1/2 or p-Smad2/3 were quantified by densitometric scanning and normalized by internal control (β-actin). CTGF and CYR61 were expressed predominantly in ESCC tissues compared with the match normal tissues.

In ESCC, THBS1, Cyr61 and CTGF immunoreactivities all displayed intense diffuse cytoplasmic staining (Figure 1A, C and 1E). In contrast, these proteins just showed weak staining in the basal and suprabasal proliferative layers of normal epithelium (Figure 1B, D and 1F). Over-expression was defined as intense diffuse cytoplasmic staining in >25% of tumor cells. Hence, 72/80, 41/80 and 39/80 for THBS1, Cyr61 and CTGF were respectively designated as over-expressions. In comparison with the staining in normal epithelial tissue, their over-expressions were all statistically significant (*P *< 0.05). In addition, elevated levels of CTGF and CYR61 protein were also found in human ESCC tissues compared with the paired normal tissue from the patients as shown by western blotting analysis (Figure 2).

### Correlations of THBS1, Cyr61 and CTGF expression with various clinicopathologic factors

To obtain a better understanding of the clinical significance of THBS1, Cyr61 and CTGF expression in ESCC, we correlated their expression with a series of clinicopathological.

As shown in Table 1, THBS1 over-expression was significantly associated with TNM stage (*P *= 0.029) and regional lymph node invasion (*P *= 0.026). No association was found between THBS1 status and the other clinicopathological variables. For Cyr61 and CTGF, there was no association between their expressions and these clinicopathological variables (Table 1).

### Impact of THBS1, Cyr61 and CTGF expressions on the overall survival of ESCC patient

**Figure 3:**
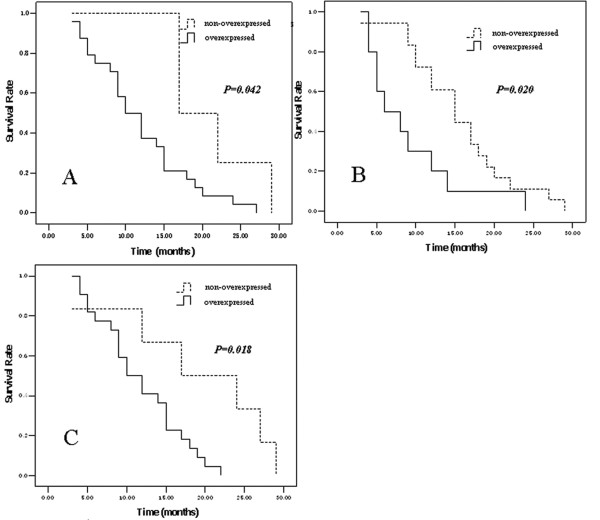
**Kaplan-Meier estimates of the survival by THBS1 (A), Cyr61 (B) and CTGF (C) status**. THBS1, Cyr61 and CTGF over-expressions all have significantly lower survival rates than their respective non-overexpression.

Survival analysis on single protein expression demonstrated that THBS1, Cyr61 and CTGF over-expressions were all associated with shorter survival (*P *= 0.042, *P *= 0.020 and *P *= 0.018, respectively) (Figure 3).

**Figure 4:**
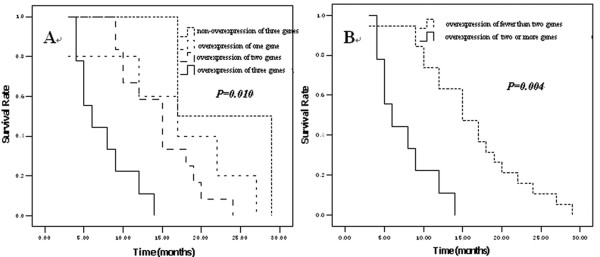
**Kaplan-Meier estimates of the survival by the combined expression of THBS1, Cyr61 and CTGF**. (A) revealed that the more overexpressed genes, the shorter the patient survival. (B) showed that patients with fewer than two overexpressed genes had longer survival.

We also explore the impact of combined expression of these genes (optional two or all three genes) on patient survival. Results revealed that combined expression of the three genes resulted in remarkable low survival. The more genes over-expressed, the shorter survival presented (Figure 4A). Obviously, ESCC patients with two or three genes over-expression had a shorter survival rate then patients with single gene over-expression (Figure 4B).

### Multivariate analysis for the prognostic value of THBS1, Cyr61 and CTGF expressions

Since THBS1, Cyr61 or CTGF expression was shown to have significant impact on the overall survival of patients with ESCC, we included the expressions of these genes in further prognostic value analysis.

Using the Cox proportional hazards model, we performed multivariate analysis to assess the independent predictive value of THBS1, Cyr61 and CTGF expressions for overall survival. The following prognostic variables were also included TNM stage, invasion degree and histological stage. Noticeably, CTGF and Cyr61 expression status could be independent prognostic factors for ESCC patients (*P *= 0.028, *P *= 0.009, respectively, Table 2 and 3) but not THBS1 (Table 4).

**Table 2:** Multivariate Cox regression analysis by Cyr61 expression levels

Variable	Hazard ratio	95% confidence interval	*P*
Regional lymph node	1.194	0.173–8.240	0.858
Primary tumor	1.346	0.352–5.137	0.664
Histologic grade	0.484	0.215–1.093	0.081
TNM Stage	1.504	0.459–4.926	0.500
Cyr61 overexpressor	5.347	1.374–20.816	0.016*

• Significant at *P *< 0.05 level

**Table 3:** Multivariate Cox regression analysis by CTGF expression levels

Variable	Hazard ratio	95% confidence interval	*P*
Regional lymph node	1.694	0.347–8.279	0.515
Primary tumor	3.839	1.016–14.506	0.047*
Histologic grade	1.418	0.556–3.616	0.464
TNM Stage	1.091	0.384–3.098	0.871
CTGF overexpressor	27.099	3.535–207.728	0.001*

* Significant at *P *< 0.05 level.

**Table 4:** Multivariate Cox regression analysis by THBS1 expression levels

Variable	Hazard ratio	95% confidence interval	*P*
Regional lymph node	0.821	0.135–4.990	0.830
Primary tumor	0.990	0.275–3.568	0.988
Histologic grade	0.553	0.242–1.267	0.161
TNM Stage	1.672	0.514–5.437	0.393
THBS1 overexpressor	3.667	0.811–16.049	0.092

* Significant at *P *< 0.05 level.

## Discussion and Conclusion

TGF-β signaling pathway is operative in some tumor cells and can contribute to tumor invasion, survival, and metastases [5]. In esophageal carcinoma, altered expression of TGF-β receptors, Smad2 and Smad4, was correlated with tumor progression or poor prognosis [18]. Recent study also revealed that the TGF-β pathway was disturbed in ESCC and the expression of TGF-β1 was up-regulated, which might be responsible for the high invasive ability of ESCC cells [19-21]. In this study, we further explored the expression and prognostic significance of three TGF-β-relative genes (THBS1, CTGF and Cyr61) in ESCC.

Cyr61 and CTGF, both transcriptional targeted gene of TGF-β pathway, are members of the CCN (Cyr61/CTGF/NOV) family. The altered-expressions of CCN proteins had been reported in a wide range of tumors such as breast cancer, non-small lung cancer and glioma [22-25]. Studies also suggest that expression and role of the CCN proteins is dependent on the tumor type in which they interact with different cell surface receptors [11]. In the present study, by employing immunostaining and western blotting analysis, we first confirmed the over-expression of CTGF in ESCC and also revealed that Cyr61 in ESCC was markedly higher than that in normal esophageal tissue. Furthermore, we showed that over-expression of CTGF or Cyr61 was related to poor survival and Cyr61 and CTGF were independent prognostic factors in ESCC. CTGF and Cyr61 were reportedly related to poor survival of patients in several cancers such as lung cancer and endometrial cancer [26,27]. In ESCC, however, a previous study suggested that high CTGF mRNA levels in ESCC were associated with longer survival, which seemed inconsistent with our findings [16]. Possible explanation for the above contradiction might be as follow: in their study, the mRNA was extracted from the whole cancer tissue which included mRNA of cancer cells as well as some stromal cells; but in this study, we aimed directly at the protein expression level of CTGF in the cancer cells. Study for the more precise mechanism is underway.

The expression of THBS1 is capable of activating TGF-β signal pathway, leading to transcription of Cyr61 and CTGF [7,8]. Methylation-induced silencing of THBS1 expression correlated with impaired TGF-β signaling as indicated by decreased Smad2 phosphorylation and nuclear localization [28]. THBS1 up-expression has been found in metastatic lesions of colon tumors and osteosarcoma and was significantly associated with overall survival in bladder cancer and lymph node metastasis in gallbladder adenocarcinoma [29-31]. Recently, it is reported that loss of THBS1 expression was also significantly associated with distal location, vascular invasion and distant metastasis in gastric cancer and with unfavourable histological grade and shorter overall survival in penile squamous cell carcinoma [32,33]. These reports suggested that THBS1 played diverse roles in the tumorigenesis. Possible mechanism is that there are two temporally distinct phases to the effect of THBS1 on cancer progression. During the early stage, THBS1 inhibits neovascularization and holds tumor growth. In latter stage, THBS1 may function as an adhesive protein or a modulator of extracellular proteases to promote tumor invasion [34]. Indeed, in our study, THBS1 was overexpressed and correlated with regional lymph node invasion in ESCC and, interestingly, patients with two or more of THBS1, Cyr61 and CTGF over-expressions had significantly shorter survival. Therefore, we postulated that overexpression of THBS1 might lead to more interaction with LTBP in the upstream of TGF-β pathway contributing to the activation of TGF-β signaling pathway in ESCC.

In conclusion, we showed here, three TGF-β-related genes (CTGF, CYR61 and THBS1) were over-expressed in ESCC. THBS1, Cyr61 and CTGF might be significant diagnostic markers for ESCC. More importantly, Cyr61 and CTGF could serve as independent prognostic markers for ESCC. Further studies will be needed to establish the biological significance of THBS1, Cyr61 or CTGF expression and to examine the possibility as therapeutic targets in ESCC.

## Competing interests

The authors declare that they have no competing interests.

## Authors' contributions

ZQZ, JJX, ZYS, QYZ, JHS, LYX and EML conceived the study, participated in its design, coordination and in the draft of the manuscript. CWH, JL and JHS provided clinical samples and background. ZQZ, QYZ, JJX and LYX contributed to the statistical analysis. ZYS and LYX supervised the sample preparation and protein extraction; ZQZ, JJX, ZYS and LYX performed the immunohistochemical evaluation and western blotting analysis; all authors have read and approved the final manuscript.

## Pre-publication history

The pre-publication history for this paper can be accessed here:

http://www.biomedcentral.com/1471-2407/9/291/prepub
